# Dislocation Densities and Velocities within the *γ* Channels of an SX Superalloy during In Situ High-Temperature Creep Tests

**DOI:** 10.3390/ma11091527

**Published:** 2018-08-24

**Authors:** Thomas Schenk, Roxane Trehorel, Laura Dirand, Alain Jacques

**Affiliations:** 1 Institute Jean Lamour, CNRS UMR 7198, 54011 Nancy, France; roxane.trehorel@univ-lorraine.fr (R.T.); alain.jacques@univ-lorraine.fr (A.J.); 2 Laboratory of Excellence on Design of Alloy Metals for low-mAss Structures (DAMAS), Université de Lorraine, 57073 Metz, France; 3 Fives Stein Bilbao, S.A., 48011 Bilbao, Spain; laura.dirand@gmail.com

**Keywords:** nickel-based single crystal superalloy, lattice mismatch, in situ experiments, X-ray diffractometry, creep

## Abstract

The high-temperature creep behavior of a rafted [001] oriented AM1 Ni-based single crystal superalloy was investigated during in situ creep tests on synchrotrons. Experiments were performed at constant temperatures under variable applied stress in order to study the response (plastic strain, load transfer) to stress jumps. Using two different diffraction techniques in transmission (Laue) geometry, it was possible to measure the average lattice parameters of both the γ matrix and the γ′ rafts in the [100] direction at intervals shorter than 300 s. The absolute precision with both diffraction techniques of the constrained transverse mismatch (in the rafts’ plane) is about 10^−5^. After stress jumps, special attention is given to the evolution of plastic strain within the γ channels. The relaxation of the Von Mises stress at leveled applied stress shows evidence of dislocation multiplication within the γ channels. From the analysis, we showed an interaction between plastic stress and dislocation density of the γ phase.

## 1. Introduction

Ni-based superalloys are used primarily for manufacturing turbines in power plants and jet and helicopter engines [1]. Their high creep resistance is mainly due to precipitation hardening of the solid solution fcc γ phase by L1_2_ γ′ precipitates [2]. The best creep resistance therefore shows single crystals being used for parts exposed to very high temperatures and stresses.

Under these conditions, cuboidal γ′ precipitates evolve into semi-coherent platelets embedded in the softer γ matrix. This results in a quasi-lamellar structure perpendicular to the applied tensile stress $\sigma_{a}$ of alternating γ and γ′ successive layers. This directional coarsening process is widely known as rafting [3,4,5,6].

At high temperatures the hard γ′ phase deforms by dislocation climb [7,8,9], while the softer γ channels are plastically deformed by a glide of the usual fcc a/2 <110> {111} dislocations [10]. The high symmetry of the [001] tensile axis results in eight of the 12 available slip systems being equally active. However, the long-term aim of understanding the high-temperature creep behavior of superalloys remains elusive as the behavior of each phase within the rafted microstructure is different to the behavior of single crystals of the same materials and the actual stress and strain state of these phases is generally unknown.

While in the rafted microstructure we can assume that the $\sigma_{zz}$ component of the stress tensor in each phase is equal to the $\sigma_{a}$ applied stress, the $\sigma_{xx}=\sigma_{yy}$ components result from coherence stresses and from the difference in plastic strain between both phases. The coherence stresses are due to a known [11] temperature-dependent natural γ/γ′ lattice mismatch defined as:

(1) $$\delta =2\frac{a_{\gamma^{\prime }}-a_{\gamma },}{a_{\gamma^{\prime }}+a_{\gamma }}$$

where $a_{\gamma }$ and $a_{\gamma^{\prime }}$ are the free lattice parameters of the unconstrained γ and γ′ phases, respectively. As mobile dislocation segments glide within the channels of the rafted microstructure, they leave straight segments at the intersections of their slip planes and the γ/γ′ interface. These dislocations react with each other to form an interface network. This results in a constrained lattice parameter misfit perpendicular to the tensile axis (i.e., in the plane of the interfaces) $\delta_{⊥}$:

(2) $$\delta_{⊥}=2\frac{a_{\gamma^{\prime }200\;}-a_{\gamma 200}}{a_{\gamma^{\prime }200\;}+a_{\gamma 200}}=-\frac{b}{d}.$$

This so-called perpendicular misfit $\delta_{⊥}$ (typical values of −$3\times 10^{-3}$) equals to −b/d where b is the magnitude of the Burgers vector and d is the average distance between interface dislocations [12]. Depending on the experimental conditions the magnitude of $\delta_{⊥}$ can be lower than, equal to, or larger than the magnitude of δ. The stresses generated by the interface dislocation network can thus decrease, cancel out, or overcompensate for the stresses due to the natural lattice mismatch. As $\delta_{⊥}$ results from dislocations that moved within the γ channels and did not cross the interface, it is also related to the difference between the plastic strain of the two phases.

The perpendicular misfit $\delta_{⊥}$ can be measured by analyzing the diffraction profile of a (200) or (020) reflection using synchrotron X-ray beams in transmission [13]. Such measurements have been carried out by Three-Crystal Diffractometry, and also by Far-Field Diffractometry (i.e., a set-up in Laue geometry, but with the detector put at far field [14]). The high precision of the relative positions of diffraction angles (10^−5^) was possible due to these techniques. Both the (200) γ and (200) γ′ peaks can be recorded with a single scan/image in a short time span, and it is possible to follow the variations of $\delta_{⊥}$. Using a multilayer model, thoroughly detailed in [15], we can calculate the thermomechanical response of both phases to changes in loading or temperature conditions [15,16,17,18,19,20,21].

The aim of the experiments described below was to study the effect of the size of the microstructure (thickness of a *γ* channel + thickness of a *γ*′ raft) on the mechanical behavior and to use a new diffraction technique with an improved time resolution in order to follow fast transients (a few hundred seconds) in the behavior of the rafts. However, in this paper we shall focus on the behavior of the γ channels. We use the variations with time of $\delta_{⊥}$ during in situ, high-temperature mechanical tests with stepwise loading to show evidence of dislocation multiplication within the γ channels and build a constitutive law for the γ channels.

## 2. Materials and Methods

### 2.1. Materials

The experiments were performed using specimens of the AM1 Ni-based single crystal superalloy provided by SNECMA-SAFRAN group (Courcouronnes, France) and by ONERA (Châtillon, France) [22]. Specimens having a [001] orientation within a few degrees were machined out from single crystalline AM1 rods. The samples diameters for samples were chosen as a function of the used X-ray energies to obtain the optimum ratio of diffraction intensity versus X-ray absorption. TCD samples have been submitted to a “*standard*” heat treatment [23], a solution treatment at 1300 °C for 3 h and air quenched with a speed of 10 °C per second, followed by two aging steps. This leads to a structure of well-defined cuboidal regularly arranged γ′ precipitates with an average edge length in the 0.4–0.5 µm range and a maximum creep life. TCD samples are of 3.4 mm in diameter and 29 mm in gauge length.

**Figure 1:**
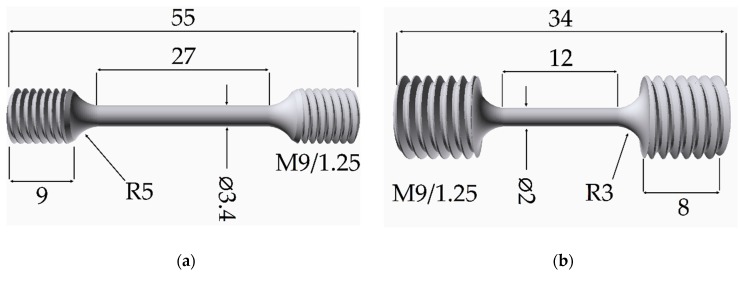
TCD specimen (**a**) and Far-Field Diffractometry specimen (**b**).

Samples for Far Field Diffractometry are 2 mm in diameter and 12 mm in gauge length. Both sample geometries are depicted in Figure 1 and verify the DIN 50125 standard [24], since the parallel length of the sample is ≥6 times the diameter, and the neck radius is larger than 0.75 times the diameter of the respective specimen. To obtain a different periodic γ/γ′ height of the “*lamellar*” microstructure, casted AM1-samples just annealed at 1300 °C for 3 h and air quenched with a speed of 10 °C per second, but not aged. The resulting cubic γ′ precipitates are more disordered and have an average edge length of 0.25 µm. After rafting, the mean value of the period (sum of the height of one raft and one channel) is lower (623 nm) than that of the “*standard*” heat treatment (893 nm).

These values were measured for a “*standard*” specimen crept at 1000 °C (TCD measurements, on BW5, HASYLAB, Hamburg, Germany) and a “*small*” microstructure specimen crept at 970 °C (Far-Field Diffractometry, on ID11, ESRF, France), which we shall mainly use for illustration.

### 2.2. High-Temperature Straining Device

The specimens were tested in a ”home-made” high-temperature straining device [25]. The sample is radiation-heated by two separate heating zones in a vacuum. The combination of radiation heating and heat losses (via the water-cooled rods, for instance) leads to a near-constant (slightly parabolic) temperature distribution within 5 mm of the sample center. The elongation of the sample is measured by a LVDT extensometer with a resolution of 0.5 μm and the applied force is recorded by a 5 kN force cell.

**Figure 2:**
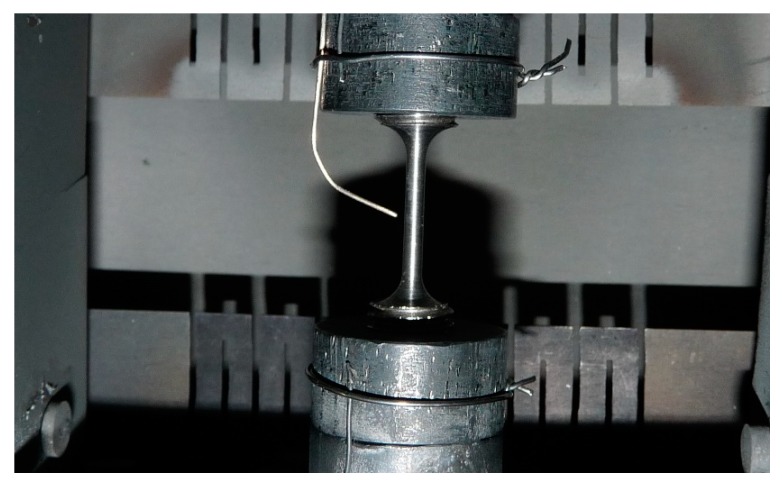
Position of the three thermocouples.

Temperatures are measured with 3 K-type thermocouples: As shown in Figure 2, two are positioned on the upper and lower grip, and are for temperature regulation, and the third is on the center of the sample. A stepper motor with a high gear ratio is coupled to the mobile rod, driving the upper grip of the straining device. All parameters (heating, force, strain rate of the stage) are continuously recorded via a LabVIEW^TM^ program.

The parabolic temperature profile is systematically checked by diffraction at the beginning of each test. The beam position along *z* corresponds to the hottest part (lowest 𝛩*_B_*) of the specimen.

### 2.3. In Situ XRD

TCD and Far-Field Diffractometry have been thoroughly compared in [14,26].

For in situ creep tests (in the ”home-made” high-temperature straining device), the bulk sample is placed with the high-temperature straining device on the diffractometer. The monochromatic beam travels through the specimen and the single crystal samples are rotated along (the vertical [001] tensile axis) to bring the (200) lattice plane into Bragg condition.

**Figure 3:**
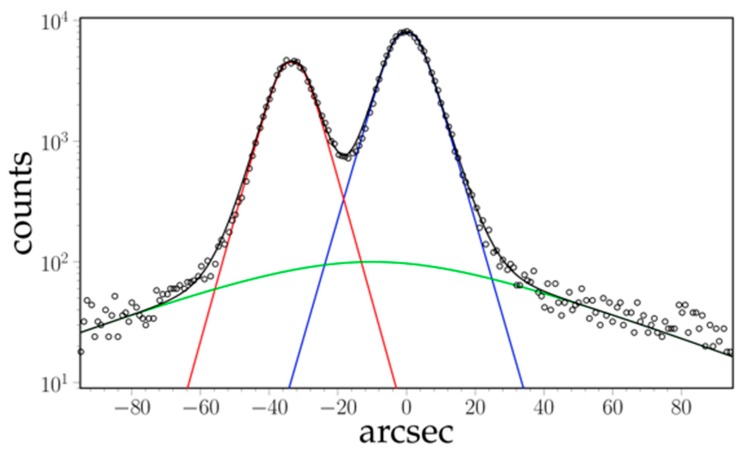
Typical X-ray data and fit of TCD intensity profile for the two peaks of phases γ  (red) and γ′  (blue).

TCD experiments using three crystals in a non-dispersive (+, −, +) geometry were done at 120 KeV ($\lambda =1.033\times 10^{-11}\;$ m) at DeSy BW5 [13] using an energy selective Ge detector. A 1D intensity profile of a (200) peak scan of the “*standard*” sample (Figure 3) is typically obtained in 300 s.

Furthermore, we investigated creep of samples using Far-Field Diffractometry at ESRF ID11, at 67 KeV ($\lambda =1.851\times 10^{-11}\;$ m) with a 2D 2048 × 2048 pixel FReLoN Camera with a pixel size of 50 µm × 50 µm as detector. The camera was placed (as far as possible) at 8.5 m from the specimen. From the recorded images, we obtained 1D integrated intensity profiles along 2θ by adding up the pixels’ intensities of the detector along the direction perpendicular to the diffraction plane. Acquisition times for a (200) peak using Far-Field Diffractometry is reduced to seven seconds permitting to follow fast transients.

For both experiments the gauge volume is large enough to probe both dendritic and interdendritic zones.

In Figure 3 a typical TCD intensity plot is shown together with the profile fitting functions. All diffraction profiles (independently of the applied diffraction set-up) were fitted as the sum of three symmetric peaks, corresponding to the γ′ rafts (blue in Figure 3), the γ channels (red in Figure 3), and a wide tail for the background (supposed to be the contribution of the distorted zones near dislocation cores).

An automated procedure using a dedicated ad hoc fitting function (Equation (3), [18]) for both γ and γ′ peak-shapes is used:

(3) $$P_{i}\left(\alpha \right)=A_{i}\exp[1+{\left[1-{\left(\frac{\left|\alpha -\;\alpha_{0i}\right|}{w_{i}}\right)}^{\beta_{i}}\right]}^{\frac{1}{\beta_{i}}}].$$

The fit parameters are the peak positions $\alpha_{0i}$, the peak heights $A_{i}$, powers $\beta_{i}>2\;$ that define the sharpness of the peaks, and the profile parameters $w_{i}$ defining the slope of Napier logarithm of the peaks. The centers of the fitted γ and γ′ peaks are used to calculate the perpendicular misfit $\delta_{⊥}$, and the area under the peaks can be used to evaluate the phase fraction $f_{XRD}^{\prime }$ of the γ′ rafts (assuming the same structure factors for both phases).

### 2.4. Post Mortem TEM and SEM Studies

**Figure 4:**
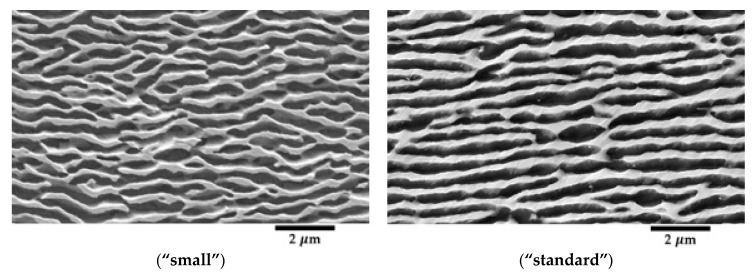
SEM micrographs of two specimens with different initial microstructure at different temperatures: 970 °C (“**small**” microstructure), 1000 °C (“**standard**”).

At the end of the in situ X-ray experiments, crept samples were cooled down fast, cutting the heating under the load to freeze their microstructure (thickness of rafts and channels, dislocation densities). TEM (FEI/Philips CM200, Eindhoven, The Netherlands) please add the information and SEM (Philips/FEI XL30 S-FEG, Eindhoven, The Netherlands) micrographs were taken of both samples. All samples exhibit a classical rafted microstructure (Figure 4). The thicknesses of channels and rafts were measured with an intercept method.

Since the equilibrium phase fraction f′ of $\gamma^{\prime }$ at room temperature is larger than at high temperatures, small $\gamma^{\prime }$ cubes precipitate within the γ-channels during cooling. The γ/γ′ interfaces are also expected to migrate during cooling. The thicknesses of the channels measured *post mortem* samples thus slightly underestimate its value at high temperature. However, the average period of the microstructure (one raft and one channel) does not change during cooling.

### 2.5. Data Analysis

The plasticity of the γ-phase (matrix) of an AM1 superalloy is studied by evaluating stress/plastic strain curves obtained by similarly following temperature, total elongation, and (2D) diffraction patterns of the (200) γ/γ′ planes. The raw relative precision in peaks shifts due to changes in temperature or stresses, as well as the difference of the parameters of γ and γ′ peaks after fitting the intensity profiles is $10^{-5}$, much better than the precision of the applied force, the temperature, and the elongation of the specimen.

Assuming that the specimen’s volume remains constant during plastic strain we correct the applied stress $\sigma_{a}$ using the total plastic deformation $\varepsilon_{zz,tot}^{pl}$ of the sample:

(4) $$\sigma_{a}=\left(1+\varepsilon_{zz,tot}^{pl}\right)\frac{F_{a}}{S_{0},}$$

where $F_{a}$ is the applied force and $S_{0}$ the initial section of the specimen.

The evolution of the lattice parameters in the [100] direction allows us to deduce the elastic strains of the *γ* channels and the *γ*′ rafts and also the average elastic strain in the [100] direction. With the multilayer model given in [15], it is possible from this recorded creep data to calculate with a good precision both the main components of the stress tensor for each phase and the strain of each phase, provided that the actual value of the perpendicular misfit $\delta_{⊥}$ is known.

The following approximations are used:

- homogenous and isotropic phases
- same Poisson coefficient ν=0.42 [4]
- distinct Young’s moduli:  E (γ), E′ (γ′)
- symmetric stress tensors (i.e., $\sigma_{xx}=\sigma_{yy}$).

The transverse stress $\sigma_{xx}$ within the channels is given by [15]:

(5) $$\sigma_{xx}=-\frac{\chi E}{1-\nu }\left[\;\left(\delta_{⊥}-\delta \right)+\left(\frac{1}{E^{\prime }}-\frac{1}{E}\right)\nu \sigma_{a}\right]=A\cdot \left(\delta_{⊥}-\delta \right)-B\cdot \sigma_{a},$$

(6) $$\mathrm{where}\;\chi =\frac{E^{\prime }f\prime \;}{E\left(1-f\prime \right)+E^{\prime }f\prime }.$$

Equation (5) allows us to calculate the von Mises stress:

(7) $$\sigma_{VM}=\sigma_{a}-\sigma_{xx},$$

which is a function of parameters $\left(\nu ,\sigma_{a},f^{\prime },\;\delta_{⊥}-\delta ,E,E^{\prime }\right)$. ν,δ are known, $\delta_{⊥},\;f^{\prime }=f_{XRD}^{\prime }$ can be measured and the evolution of $E,E^{\prime }$ is given in [15].

The plastic strain of the whole specimens $\varepsilon_{zz,tot}^{pl}=\left(1-f^{\prime }\right).\varepsilon_{zz}^{pl}+f^{\prime }.\varepsilon \prime_{zz}^{pl}$ was deduced from the variations of $\Delta 𝓁/{𝓁}_{0}$ of the instantaneous elastic variation during loading and unloading at constant temperature. Still assuming a constant volume and as discussed above, the perpendicular misfit $\delta_{⊥}=\left({\varepsilon^{\prime }}_{zz}^{pl}-\varepsilon_{zz}^{pl}\right)/2$ resulting from the difference of plastic strain between both phases, we obtain the strain of the γ channels as follows:

(8) $$\varepsilon_{zz}^{pl}\approx \varepsilon_{zz,tot}^{pl}-2f\prime \;\delta_{⊥}.$$

## 3. Results

### 3.1. Real-Time Experiment with Far-Field Diffractometry

**Figure 5:**
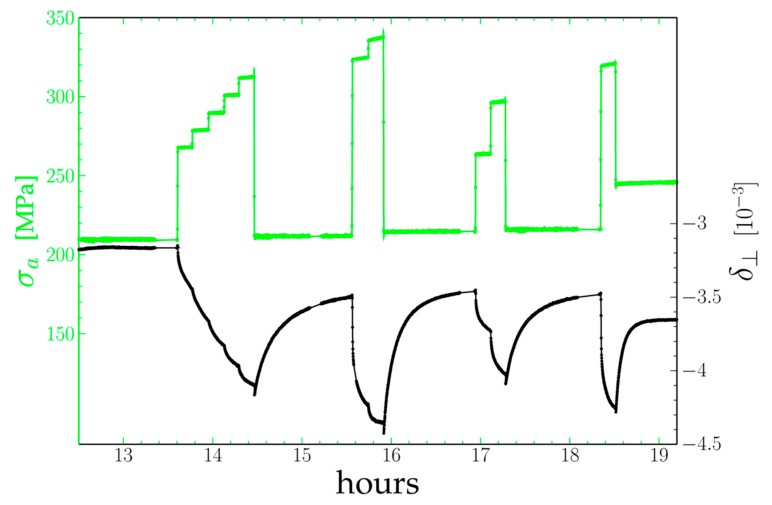
Evolution of the perpendicular misfit $\delta_{⊥}$ (black) with the corrected applied stress $\sigma_{a}$ (green) of the “small” AM1 sample at 970 °C. Each dot corresponds to a seven seconds diffraction peak recording.

The aim of the experimental procedure described below was first to obtain well-rafted microstructures with a different wavelength, then to study the response of both phases to changes in their stress state. Oriented coalescence of the *γ*′ precipitates (i.e., rafting) results from the difference in stored elastic energy between the different *γ* channels in the initial cuboid microstructure [5,6]. In superalloys with a negative lattice mismatch, during tensile creep tests, the ‘horizontal’ channels perpendicular to the tensile axis are plastically strained while the ‘vertical’ channels remain nearly dislocation-free. As the coherence stresses are partly relaxed by plastic strain in the ‘horizontal’ channels their stored energy becomes lower than that of the ‘vertical’ channels: the horizontal channels widen, while the vertical channels become thinner and disappear. The pre-strain load must be chosen so that dislocations can glide within all the horizontal channels, i.e., the total stress on dislocations (applied stress + coherence stresses) must be larger than the Orowan stress. As the Orowan stress increases when the thickness of the channels decreases (about 160 MPa for the “*small*” microstructure vs. 95 MPa for the standard one), the pre-strain load was increased from 150 MPa to 200 MPa. As the horizontal channels widen during rafting, the final Orowan stress is lower. Last, former experiments [15] showed a strong dependence of the strain rate of the *γ*′ rafts vs. the internal stress $-\sigma \prime_{xx}$. In order to investigate this behavior and its dependence on the microstructure, the experimental conditions were chosen so that $-\sigma \prime_{xx}$ would vary between 0 MPa and about 100 MPa. The specimen with an initial “*small*” microstructure was first heated to 970 °C, then put under a 200 MPa load. The (200) diffraction profile was continuously followed during the test. It evolved from the one peak distribution (mainly γ′) with a bump on the lower 2θ side (mainly γ) observed for the cuboids microstructure [17] into a well-defined two peaks profile as seen in Figure 3 for a rafted microstructure. After 13 h the rafting was deemed to be complete, and the tests began using the same procedure as in [15] (Figure 5). The specimen was submitted to a stepwise loading first to 260 MPa, then by 10 MPa positive steps up to 300 MPa (each step lasting 10 min), then unloaded to 200 MPa (corresponding to a corrected applied stress of 212 MPa). Those first loadings are reported in Figure 4a with greater detail). After a ~1 h relaxation further upwards and downwards steps under different loads followed by a relaxation were done. The specimen was then cooled down to room temperature under a 230 MPa stress.

At each positive stress jump both γ and γ′ peaks shift to a larger 2θ angle by the same amount ($\delta_{⊥}\;$ does not change.), as the transverse lattice parameters of both phases decrease instantaneously according to the material’s Poisson’s ratio: This is the elastic response. Following this shift, the distance between both peaks increases rapidly, then at a slower pace: $\delta_{⊥}\;$ becomes increasingly negative. Each downwards stress jump first results in a dip of $\delta_{⊥}$: as the length of the tensile rod decreases the specimen moves downwards along the z axis by a few tens of micrometers and the specimen’s volume illuminated by the 50 × 50 µm^2^ X-ray beam changes (This effect is also present during positive steps but is masked by the fast decrease of $\delta_{⊥}$). The misfit then increases in algebraic value until it settles after one hour.

**Figure 6:**
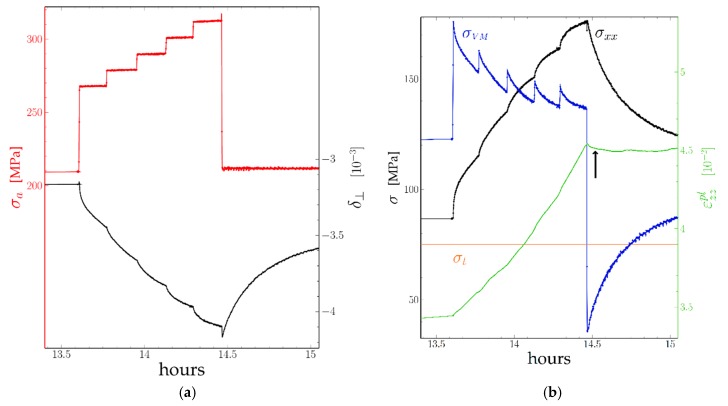
Detail between 13 h 30 and 15 h of the creep test of a sample with an initial “**small**” microstructure at 970 °C: (**a**) applied stress $\sigma_{a}$ (red), $\delta_{⊥}$, (black), **b**) $\sigma_{xx}$ (black) $\sigma_{VM}$ (blue) and plastic strain $\varepsilon_{zz}^{pl}$ in green.

At each measurement point the $\sigma_{xx}$ stress component has been calculated using Equation (5), then the Von Mises stress $\sigma_{a}-\sigma_{xx}$. The plastic strain was obtained using Equation (8). Both are plotted on Figure 6b. As it can be seen, each upwards step of the applied stress results in an equivalent step in the Von Mises stress. The Von Mises stress then decreases first fast, then more slowly as the plastic strain of the rafts increases, $\delta_{⊥}$ decreases algebraically, and the $\sigma_{xx}$ stress component (i.e., the back stress due to interface dislocations) increases: The Von Mises stress is obviously larger than the Orowan stress. The opposite is observed after a downwards stress step, but as the plastic strain of the channels decreases only slightly the increase of $\delta_{⊥}$ results from the plastic strain of the rafts. Following a downwards stress step, the strain rate of the channels is first negative (arrow), then zero, and later becomes positive even before the next positive stress jump.

An interesting point is the evolution of the stress and strain rates during the initial stepwise loading between 13 h 30 and 14 h 30. While each step results in an increase then a decrease of the Von Mises stress, the global tendency is a decrease of the Von Mises stress. On the contrary, the plastic strain rate increases. This point will be discussed below.

### 3.2. Successive Relaxation Tests

**Figure 7:**
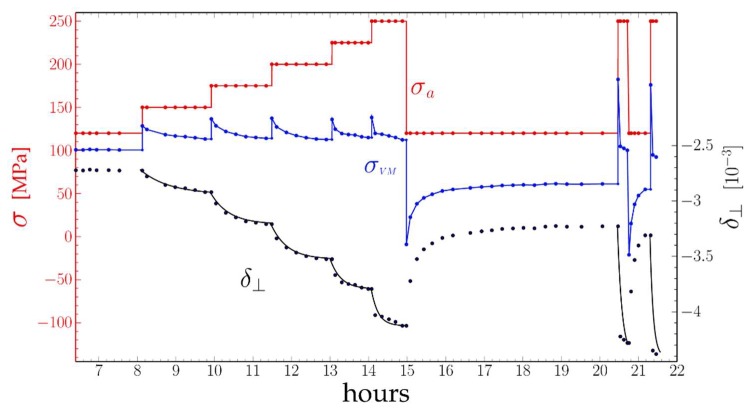
Creep test of a “standard” sample at 1000 °C: applied stress $\sigma_{a}$ (red), calculated $\sigma_{VM}$ (blue) and in black, $\delta_{⊥}$ measured (points) and calculated (lines) for an exponential decay of $\delta_{⊥}$ for the different decay times given in Table 1.

Figure 7 shows the evolution of the Von Mises stress of a “**standard**” AM1 sample recorded by Three-Crystal Diffractometry (with peak recording lasting 300 s instead of 7 s).

The same procedure as before was qualitatively followed: heating to 1000 °C then loading to 120 MPa until complete rafting, and stepwise loading and unloading. The evolution of the applied stress $\sigma_{a}$, the perpendicular misfit $\delta_{⊥}$ and the calculated Von Mises stress $\sigma_{VM}$ are shown in Figure 7. The general shape of these functions follows the same behavior as that described in Section 3.1 with some differences detailed below.

The first five 25 MPa stress jumps at levels from 120 MPa up to 250 MPa were performed within 7 h. During the one-hour span following a loading step, $\delta_{⊥}$ had time to settle at increasingly negative levels and the Von Mises to return each time to a 113 MPa level. The strain rate of the channels, as deduced from the leveling of $\delta_{⊥}$, then fell to 0. The evolution of $\delta_{⊥}$ was approximately exponential, but as can be seen from the figure the evolution was initially quite slow then increasingly fast: the characteristic relaxation times $\tau_{i}$ decrease with each step i.

## 4. Analysis of the Experimental Results

### 4.1. Base of Modelling

The strain rate ${\dot{\varepsilon }}_{p}$ of a material is commonly given by the Orowan equation:

$${\dot{\varepsilon }}_{p}=s.\rho_{m}.b.v,$$

where *s* is the Schmid factor (here: $1/\sqrt{6}$), $\rho_{m}$ the density of mobile dislocation segments, b the magnitude of the Burgers vector, and v the velocity of the dislocation segments. In the following, we shall try to separate the respective contributions of the density and the velocity of dislocations.

Dislocation glide in γ channels will take place, if the Von Mises stress $\sigma_{VM}$ is large enough to overcome a threshold stress stress $\sigma_{t}$, generally assumed to be the Orowan stress. The glide velocity v of the dislocations should be a unique increasing function of the effective resolved shear stress $\tau^{*}$ they can feel:

(9) $$v=f\left(\tau^{*}\right)=f\left(\frac{1}{\sqrt{6}}\left(\sigma_{VM}-\sigma_{t}\right)\right)for\;\sigma_{VM}>\sigma_{t}.$$

The Orowan stress [11] results from the necessity for a dislocation segment to leave one segment at each γ/γ′ interface on both sides of a channel. If the Von Mises stress becomes lower than the threshold stress the moving segment may bow in the opposite direction, and perhaps move backwards on a limited distance by reabsorbing these segments. However, as these are supposed to climb within the interface and react with the preexisting network of dislocations [27], only a short length can be reabsorbed. Further backwards glide would require the creation of new interface dislocations with the opposite sign, i.e., a Von Mises stress with the opposite sign and a magnitude larger than the threshold stress. As can be seen from our measurements (Figure 6b), the backwards strain is quite limited (in the $5\times 10^{-4}$ range).

We may remark that as different channels have different thicknesses they also have different Orowan stresses. However, the plastic strain should stop when the local Von Mises stress (the applied load minus the local $\sigma_{xx}$ stress due to the local density of interface dislocations) is equal to the local Orowan stress. One hour after a first step, as in Figure 7, the local equilibrium has been reached for all channels, and Equation (9) will remain correct at the local level.

### 4.2. Determination of the Threshold Stress

**Figure 8:**
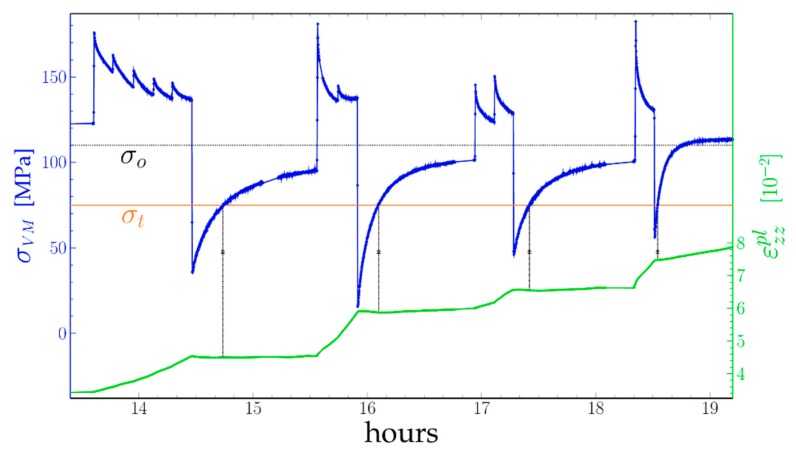
Creep test of the AM1 sample with short period at 970 °C $\sigma_{VM}$ stress (blue), expected Orowan stress $\sigma_{O}$ (black), apparent threshold stress $\sigma_{t}$ (orange) and plastic strain $\varepsilon_{zz}^{pl}$ in green.

In Equation (9) we define the Orowan stress as the stress for which the dislocation velocity is zero and above which it is positive. In the case of the TCD tests (Section 3.2) this is obviously the 113 MPa level at which the Von Mises settles after a load step. An estimation of this stress in [11], taking into account the average thickness of the channels and the elastic constants determined at high temperature indeed gives about 100 MPa. The case of the Far-Field Diffractometry test (Section 3.1) is more complicated, as the relaxation of the Von Mises stress following a load step is never complete. However, during a downwards load step, the Von Mises stress decreases by the same amount and the strain rate becomes negative (Figure 6b and Figure 8). As the Von Mises stress re-increases the strain rate becomes positive again. However, following a downwards step the Von Mises stress increases, while the strain rate becomes positive. If we determine the moment at which this change of sign takes place then look at the value of the Von Mises stress at this moment, we obtain 75 MPa. (It may be noticed that, without the applied load correction (Equation (4)) the result would have been decreasing stress.)

This 75 MPa threshold stress $\sigma_{t}$ is much lower than the expected Orowan stress, being evaluated to 101 MPa for this “**small period**” sample if compared to $\sigma_{O}$ of the “standard” sample. We can exclude such a large error in the measurement of $\sigma_{VM}$. Hence $\sigma_{t}$ cannot be $\sigma_{O}$ and we expect a different deformation mechanism than pure Orowan-limited dislocation glide for those low stresses.

### 4.3. Dislocation Velocities and Densities

**Figure 9:**
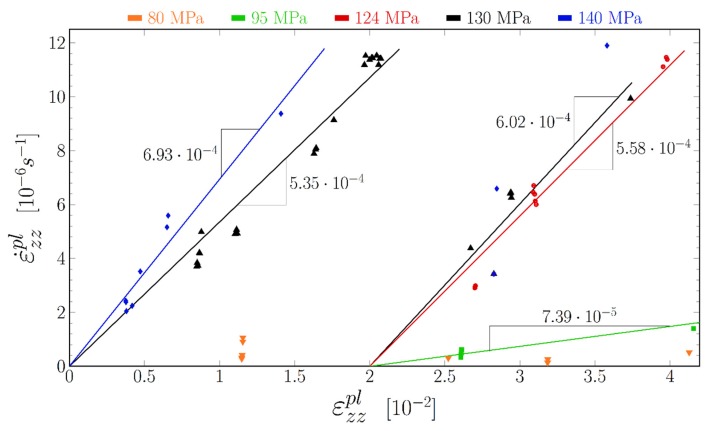
Strain rate of the γ channels as function of their strain for equal Von Mises stresses.

During the successive relaxations of Figure 8 the Von Mises stress had several times the same value. At these points, the velocity of dislocations should be the same. However, as can be seen in Figure 9, the strain rate changes.

In Figure 9, we plot the value of the strain rate for different levels of the Von Mises stress as a function of the plastic strain. As it can be seen, the points fall along straight lines. The slope S of these lines (obtained by linear regression) increases with the level of the Von Mises stress. The first two lines (140 MPa and 130 MPa, measurements between 13 h 30 and 14 h 30) intersect near the origin of the plot, while the latter lines (130 MPa, 124 MPa and 95 MPa for measurements after 16 h) intersect the plastic strain axis at $\varepsilon_{zz}^{pl}\approx 0.02$. If we admit that the dislocation velocity was the same for all points along a line, the only explanation is that the dislocation density increases linearly with the strain, and the initial dislocation density (at the beginning of the test) was quite low. The shifted lines could then be explained by a drop of the dislocation density, probably at (15 h 55) the instant of the deepest drop of the Von Mises stress during the test.

We can thus rewrite the Orowan law as:

(10) $$\dot{\varepsilon_{zz}^{pl}}=S\left(\sigma_{VM}\right)\cdot \left(\varepsilon_{zz}^{pl}-\varepsilon_{0}^{pl}\right).$$

The constant $\varepsilon_{0}^{pl}$ would be very small before (15 h 55), and 0.02 afterwards. The slope $S\left(\sigma_{VM}\right)$ should then be proportional to the dislocation velocity and should be a unique function of the Von Mises stress.

**Figure 10:**
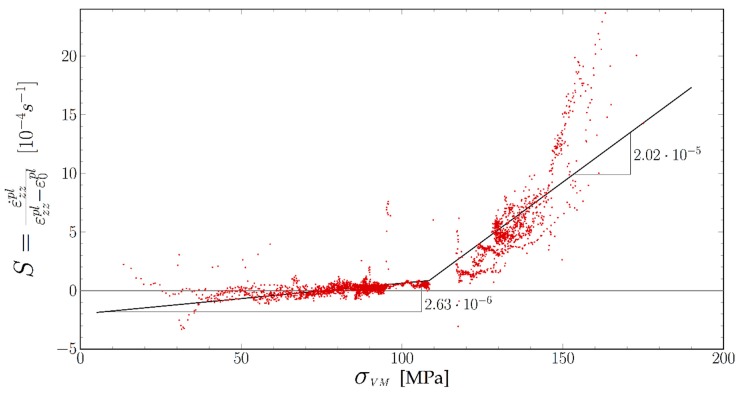
Strain rate divided by strain as function of Von Mises stresses.

In Figure 10 we plot the ratio between the plastic strain rate and the $\varepsilon_{zz}^{pl}-\varepsilon_{0}^{pl}$ factor (i.e., $S\left(\sigma_{VM}\right)$) as a function of the Von Mises stress. Despite some time-averaging (60 s) there is some scatter in the data, especially for very low stresses (<50 MPa) and very high stresses. As stated above this noise results mainly from the noise in the measure of the specimen elongation. The points at the lowest and highest stresses were recorded during specimen loading or unloading when we expected the largest errors on our experimental data. However, the whole set of points clearly settles along two lines (black). At low stresses (from 50 to 110 MPa) the behavior is linear, with a $2.3\times 10^{-6}\;$ s^−1^·MPa^−1^ slope. The best fit goes through the stress axis for $\sigma_{t}$ = 78 MPa. All these low stress points were recorded within the hour following a load drop, as the Von Mises stress was increasing again. The high Von Mises stress points (>120 MPa) were recorded during the stepwise upwards loading. They follow a line with a $2.02\times 10^{-5}\;$ s^−1^·MPa^−1^ slope. The transition between these two behaviors cannot be seen as we lack data points in the 110 MPa < $\sigma_{VM}$ < 120 MPa domain.

**Figure 11:**
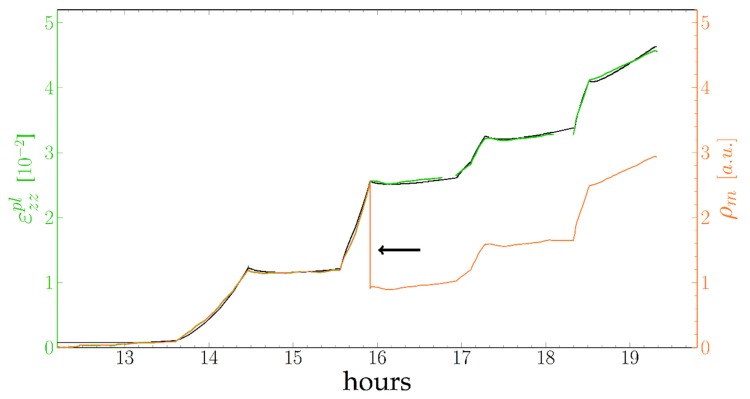
Plastic strain measured (green) and calculated (black) and dislocation density (orange). Arrow: drop of the dislocation density at 15 h 55.

In order to check the accuracy of the above modelling, we performed a simulation of the evolution of the plastic strain during the whole test. The cumulative plastic strain was calculated by integrating stepwise for an integration time of 10 s the strain rate and the plastic strain, using the two slopes S-factor adjusted from Figure 10, and an initial strain-rate of 0 before 13 h. Each time segment between a load drop and the next was taken independently, with the same $S\left(\sigma_{VM}\right)$ law but different initial $\varepsilon_{0}^{pl}$ factors in order to allow for losses of dislocations during the stress drops. Such a drop of $\rho_{m}$ calculated as $\rho_{m}=\;\varepsilon_{zz}^{pl}-\varepsilon_{0}^{pl}\;$ is indicated with an arrow on Figure 11, corresponding to an increase of the $\varepsilon_{0}^{pl}$ constant of 0.02 at 15 h 55 during the most important $\sigma_{VM}$ release. The resulting plastic strain curve is compared with the experimental one in Figure 11.

As can be seen in Figure 11, the evolution of the strain is well reproduced both in the low and high Von Mises stress regimes.

### 4.4. Successive Stress Relaxations and Activation Energy

The long recording time of TCD peaks does not allow us to follow the variations of the strain rate during a single loading step as in the above paragraph. However, as a full set of data is available between 1000 °C and 1125 °C it can be used to observe variations due to temperature. The evolution of the perpendicular mismatch recorded in Figure 7 was put into equations in [28].

**Figure 12:**
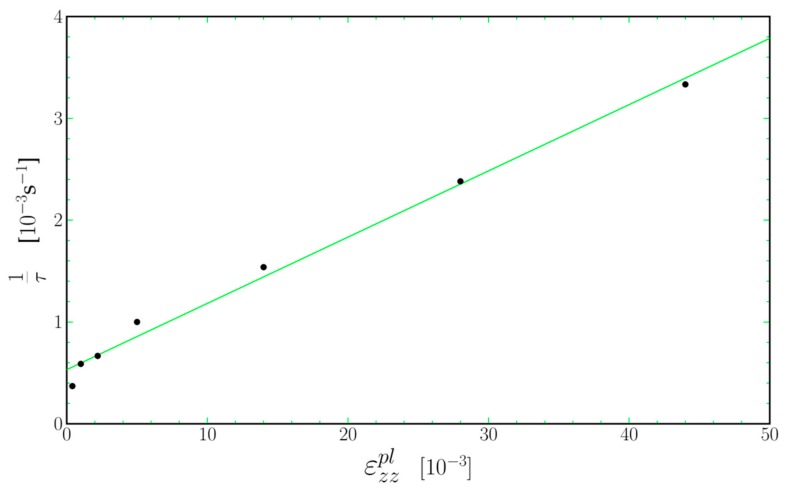
Variation of the relaxation time τ as function of the plastic deformation $\varepsilon_{zz}^{pl}$ of the γ phase during a creep test of “standard” sample.

As during the tests, the difference between the Von Mises stress and the Orowan stress varies first from zero to 25 MPa during a load step then from 25 MPa to zero during the following relaxation. The dislocations’ velocities are assumed to vary with the dislocation mobility μ as: $v=\mu \cdot \left(\sigma_{VM}-\sigma_{O}\right)$ (we neglect the low-stress regime). The relaxation time should be inversely proportional to μ and to $\rho_{m}$. The results of the successive exponential fits are given in Table 1 and Figure 12 shows the variation of $\tau^{-1}$ the inverse of the relaxation time vs. the plastic strain of the rafts $\varepsilon_{zz}^{pl}$ for the TCD specimen. As can be seen, the variation is linear with a 0.065 slope: the increase in dislocation density is proportional to the strain increment.

**Figure 13:**
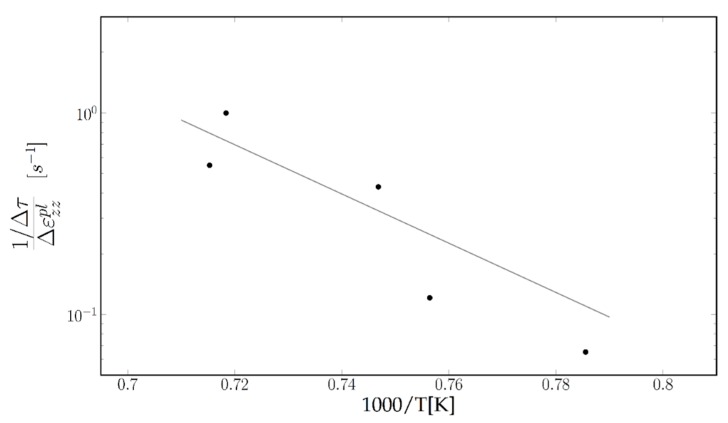
Arrhenius plot of Δ(1τ)Δεzzpl as function of the inverse temperature for deformations after stress releases following small stress jumps.

The slope should be proportional to the dislocations’ mobility μ. If we now plot the slopes obtained for different specimens at different testing temperatures, reported in Table 2 on an Arrhenius plot (Figure 13), they seem to fit on a straight line.

The corresponding activation energy is (with some incertitude) in the 3 eV range. This value is of the same magnitude as the 3.26 eV apparent activation energy given in [29] and 3.7 eV [30] for dislocation glide within the channels during Stage I of creep.

## 5. Discussion

Two points will be discussed in the following: the shape of the dislocation velocity vs. Von Mises stress (Figure 7), and the mechanisms of dislocation multiplication and annihilation.

### 5.1. Dislocation Velocity Law

Even if it gave the best fit to our data (much better than a power law or a hyperbolic sine) the apparent velocity law with two slopes and two thresholds found in Figure 10 raises a question: the 78 MPa Orowan stress does not fit with its expected value (101 MPa) and is much lower than the values found in [15], while the 108 MPa threshold is more in line with those. The strain rate of the channels measured after each unloading to 200 MPa was in the 10^−7^ range. This is low, but as the same stress level was kept for nearly one hour, the specimen elongation was about 20 µm, much larger than the error bars. Besides, the dislocation density increases slightly during that time and is consistent with that of the next high stress step: we can exclude a large error in the measurement of the strain rate.

**Figure 14:**
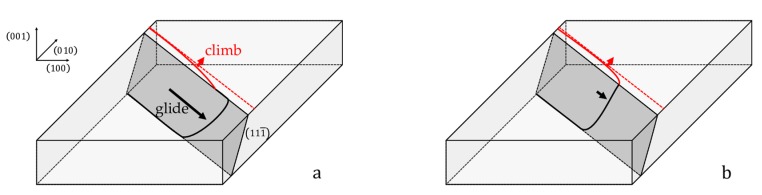
A dislocation glides within (**a**) $\left(11\bar{1}\right)$ plane of a *γ* channel leaving a dislocation segment at each intersection of its glide plane and the *γ*/*γ*′ interface. The segment at the top interface (red line) later climbs within the interface to react with another interface dislocation (red dashed line). (**b**) If the glide velocity is low the interface segment immediately bows into the interface and no extra energy is needed to create it.

The theoretical Orowan stress has been calculated assuming that a moving dislocation segment needs to leave one segment at each interface and that the work of the force on the gliding dislocation segment is equal to the energy needed to create these two dislocation segments (Figure 14a).

These segments later climb within the *γ*/*γ*′ interface until they react with other dislocations of the interface network and decrease their line energy. However, if the glide velocity of the dislocation is of the same order of magnitude as its climb velocity (Figure 14b) the interface segment can bow immediately therein: it is no longer necessary to provide its line energy.

The dislocation can thus glide slowly within the rafts under a Von Mises stress much lower than the theoretical Orowan stress. If then the Von Mises stress goes past the Orowan stress, the mobile dislocation segment can glide freely within a channel and leave the reacting segments far behind.

### 5.2. Dislocation Multiplication and Annihilation

The layered microstructure of a superalloy with a rafted microstructure presents some analogies with pearlite [31] and the Persistent Slip Bands (PSBs) observed in fcc single crystals during oligocyclic fatigue tests [32,33], and we may expect some common features in their behavior. During fatigue tests under single slip conditions, dislocations are supposed to bulge out of the PSBs upon stress reversal and screw segments to glide within the channels until they get trapped by another screw segment gliding in the opposite direction in a parallel slip plane. Both segments then annihilate by cross slip. In pearlite, a specific mechanism of dislocation multiplication (“scolopendra” sources) was observed in [31]: dislocations reaching the end of a channel between two cementite islands can propagate into neighboring channels. As in the present case we have no stress reversal and dislocations segments from the interface network are probably unable to bulge back into the channels. The multiplication mechanism should thus be of the scolopendra type. As the dislocations gliding within the channels belong to all of the eight active slip systems we also may expect different kinds of annihilation reactions to occur: reactions between dislocations of the same slip systems as in fatigue, reactions between dislocations from coplanar slip systems as well as between dislocations gliding in secant planes. However, we shall assume here that two dislocations annihilate if they pass within a distance shorter than a capture radius y of each other. (This capture distance is about 50 nanometers for screw dislocations in parallel slip planes. It should be of the order of magnitude of the channel width for dislocations moving in secant planes.) The balance equation for dislocation density as a dislocation moves by a distance dx [31] should thus be:

(11) $$d\rho =\rho \frac{dx}{L}-2y\rho^{2}dx=\frac{d\varepsilon }{sbL}-2y\rho \frac{d\varepsilon }{sb},$$

where L should be of the same order of magnitude as the rafts’ length (about 5 µm), s is the Schmidt factor for all active slip systems and b the magnitude of the Burgers vector.

As in the beginning of our experiment the initial dislocation density is low, the annihilation term should be negligible and the dislocation density proportional to the strain:

(12) $$\rho =\frac{\varepsilon }{sbL}\sim 3.10^{14}\varepsilon .$$

Using Equation (10), we thus find a relation between the dislocation velocity and factor $S\left(\sigma_{VM}\right)$:

$$v\left(\sigma_{VM}\right)=L\cdot S(\sigma_{VM}).$$

The range of dislocation velocities in the *γ* channels is $\sim 5\times 10^{-9}\;m\cdot s^{-1}$. After a 1% strain, the dislocation density within the channels should be $3\times 10^{12}{\;m}^{-2}$: this is one order of magnitude higher than actually observed by TEM [28]. However, at the end of the rafts a larger density of dislocations is observed at points where several channels interconnect: these dislocations did apparently glide along a channel and are waiting to enter the next ones. The dislocation velocity law given above is thus an average velocity law, much lower than the actual glide velocity within the channels.

These dislocations queuing up to enter the channels might also explain an apparent paradox concerning dislocation annihilation. Following Equation (11), the dislocation density should saturate for

$$\rho_{sat}=\frac{1}{2yL}.$$

Assuming the capture radius y in the 10^−7^ m range saturation should occur at $\rho_{sat}\sim 10^{12}{\;m}^{-1}$, for a 0.3% strain. This was not observed here (Figure 11). However, if the dislocation distribution is uneven, and the actual dislocation density within the channels is lower than the average density, the probability of meeting of two dislocations gliding within a channel is small and the rate of mutual annihilation lower than expected from Equation (12). Here, we have to assume that the dislocations waiting at the entry of a raft do not annihilate each other, either because their distance is larger than y or because they are bending at the entry of a channel. Under a load drop, as the Von Mises stress is near 0, these dislocations can move backwards under their mutual stress field and annihilate each other (Figure 11 at 15 h 55).

## 6. Conclusions

Using a combination of high-resolution X-ray diffraction measurements and in situ, high-temperature creep tests, it is possible to determine both the stress state and the strain rate of the *γ* channels under complicated stress paths. The main results of the analysis of experimental data are:

- The mobile dislocation density increases proportionately with the strain. This is consistent with a multiplication mechanism of the scolopendra type. However, as the dislocation distribution is uneven (there is probably a waiting time before dislocations enter the channel) usual annihilation laws do not apply and the main annihilation events take place during drops in the Von Mises stress.
- Two different velocity laws for high and low Von Mises stresses seem to operate. In the first case (Von Mises stress larger than the Orowan stress), the velocity is proportional to $\sigma_{VM}-\sigma_{O}$ and the dislocation mobility is thermally activated a ~3 eV activation energy. At Von Mises stresses lower than the Orowan stress, dislocations can still glide within the rafts without needing to trail one dislocation at each *γ*/*γ*′ interface. This mechanism is probably controlled by dislocation climb within the interface.

## Figures and Tables

**Table 1:** Adjusted parameters for calculating $\delta_{⊥}$ (Figure 12) of “standard” specimen.

Jump nº	$\sigma_{a}\;(\mathrm{MPa})$	$-\delta_{f}\;(10-3)$	$\;\varepsilon_{zz\;(10-3)}^{pl}\;$	$\;{\dot{\varepsilon }}_{zz\;(s-1)}^{\prime pl}\;$	τ (s)
1	150	2.9406	0.4	3.0 × 10^−8^	2700
2	175	3.2091	1	7.0 × 10^−8^	1700
3	200	3.52515	2.2	3.0 × 10^−7^	1500
4	225	3.79425	5	1.24 × 10^−6^	1000
5	250	4.12654	14	5.0 × 10^−6^	650
6	250	4.40814	28	1.2 × 10^−5^	420
7	250	4.40813	44	1.50 × 10^−5^	300

**Table 2:** Slope $\Delta \left(1/\tau \right)/\Delta \varepsilon_{zz}^{pl}$ for different “standard” specimens [28].

Sample	5A1	4L2	5C1	1C2	4R2
T	1000	1049	1066	1119	1125
slope	0.0651	0.121	0.43	1	0.55

## References

[1] Reed R.C., Rae C.M.F., Laughlin D.E., Hono K. (2014). 22—Physical Metallurgy of the Nickel-Based Superalloys. Physical Metallurgy.

[2] Pollock T.M., Argon A.S. (1992). Creep resistance of CMSX-3 nickel base superalloy single crystals. Acta Metall. Mater..

[3] Miyazaki T., Nakamura K., Mori H. (1979). Experimental and theoretical investigations on morphological changes of γ′ precipitates in Ni-Al single crystals during uniaxial stress-annealing. J. Mater. Sci..

[4] Pollock T.M., Argon A.S. (1994). Directional coarsening in nickel-base single crystals with high volume fractions of coherent precipitates. Acta Metall. Mater..

[5] Buffiere J.Y., Ignat M. (1995). A dislocation based criterion for the raft formation in nickel-based superalloys single crystals. Acta Metall. Mater..

[6] Véron M., Bréchet Y., Louchet F. (1996). Directional coarsening of Ni-based superalloys: Computer simulation at the mesoscopic level. Acta Mater..

[7] Srinivasan R., Eggeler G., Mills M. (2000). *γ*′-cutting as rate-controlling recovery process during high-temperature and low-stress creep of superalloy single crystals. Acta Mater..

[8] Huang M., Zhuo L., Xiong J., Li J., Zhu J. (2015). Core structure of a<100> superdislocations in a single-crystal superalloy during high-temperature and low-stress creep. Philos. Mag. Lett..

[9] Jacques A., Tréhorel R., Schenk T. (2018). High temperature dislocation climb in the γ′ rafts of Single Crystal Superalloys: The hypothesis of a control by dislocation entry into the rafts. Metall. Mater. Trans. A.

[10] Nabarro F.R.N., Frank R.N., De Villiers H.L., Heidi L. (1995). The Physics of Creep: Creep and Creep-Resistant Alloys.

[11] Jacques A., Dirand L., Chateau J.P., Schenk T., Ferry O., Bastie P. (2011). In Situ Measurement of Internal Stresses and Strain Rates by High Energy X-ray Diffraction during High Temperature Mechanical Testing. Adv. Mater. Res..

[12] Hantcherli M., Pettinari-Sturmel F., Viguier B., Douin J., Coujou A. (2012). Evolution of interfacial dislocation network during anisothermal high-temperature creep of a nickel-based superalloy. Scr. Mater..

[13] Bouchard R., Hupfeld D., Lippmann T., Neuefeind J., Neumann H.B., Poulsen H.F., Rütt U., Schmidt T., Schneider J.R., Süssenbach J. (1998). A triple-crystal diffractometer for high-energy synchrotron radiation at the HASYLAB high-field wiggler beamline BW5. J. Synchrotron Radiat..

[14] Tréhorel R., Ribarik G., Jacques A., Schenk T. (2018). Real time study of transients during high temperature creep of a Ni-based superalloy by far field high energy synchrotron X-rays diffraction. J. Appl. Crystallogr..

[15] Dirand L., Jacques A., Chateau-Cornu J.P., Schenk T., Ferry O., Bastie P. (2013). Phase-specific high temperature creep behaviour of a pre-rafted Ni-based superalloy studied by X-ray synchrotron diffraction. Philos. Mag..

[16] Royer A., Bastie P., Veron M. (1998). In situ determination of γ′ phase volume fraction and of relations between lattice parameters and precipitate morphology in Ni-based single crystal superalloy. Acta Mater..

[17] Diologent F., Caron P., D’Almeida T., Jacques A., Bastie P. (2003). The γ/γ′ mismatch in Ni based superalloys: In situ measurements during a creep test. Nucl. Instrum. Methods Phys. Res. Sect. B Beam Interact. Mater. Atoms..

[18] Jacques A., Bastie P. (2003). The evolution of the lattice parameter mismatch of a nickel-based superalloy during a high-temperature creep test. Philos. Mag..

[19] Jacques A., Diologent F., Bastie P. (2004). In situ measurement of the lattice parameter mismatch of a nickel-base single-crystalline superalloy under variable stress. Mater. Sci. Eng. A.

[20] Jacques A., Diologent F., Caron P., Bastie P. (2008). Mechanical behavior of a superalloy with a rafted microstructure: In situ evaluation of the effective stresses and plastic strain rates of each phase. Mater. Sci. Eng. A.

[21] Dirand L., Cormier J., Jacques A., Chateau-Cornu J.P., Schenk T., Ferry O., Bastie P. (2013). Measurement of the effective gamma/gamma’ lattice mismatch during high temperature creep of Ni-based single crystal superalloy. Mater. Charact..

[22] Caron P., Khan T. (1983). Improvement of Creep strength in a nickel-base single-crystal superalloy by heat treatment. Mater. Sci. Eng..

[23] Fredholm A., Khan T., Theret J.M.C.F., Davidson J.H. (1985). Alliage Monocristallin à Matrice à Base de Nickel.

[24] Testing of Metallic Materials—Tensile Test Pieces. https://www.din.de/de/mitwirken/normenausschuesse/nmp/normen/wdc-beuth:din21:262241217.

[25] Feiereisen J.P., Ferry O., Jacques A., George A. (2003). Mechanical testing device for in situ experiments on reversibility of dislocation motion in silicon. Nucl. Instrum. Methods Phys. Res. Sect. B Beam Interact. Mater. Atoms.

[26] Tréhorel R. (2018). Comportement Mécanique Haute Température du Superalliage Monocristallin AM1: Étude In Situ Par Une Nouvelle Technique de Diffraction en Rayonnement Synchrotron. Ph.D. Thesis.

[27] Epishin A., Link T. (2004). Mechanisms of high-temperature creep of nickel-based superalloys under low applied stresses. Philos. Mag..

[28] Dirand L. (2011). Fluage à Haute Température d’un Superalliage Monocristallin: Expérimentation In Situ en Rayonnement Synchrotron.

[29] Carry C., Strudel J.L. (1977). Apparent and effective creep parameters in single crystals of a nickel base superalloy-I Incubation period. Acta Metall..

[30] Ma A., Dye D., Reed R.C. (2008). A model for the creep deformation behaviour of single-crystal superalloy CMSX-4. Acta Mater..

[31] Louchet F., Doisneau-Cottignies B., Bréchet Y. (2000). Specific dislocation multiplication mechanisms and mechanical properties in nanoscaled multilayers: The example of pearlite. Philos. Mag. A Phys. Condens. Matter Struct. Defects Mech. Prop..

[32] Essmann U., Mughrabi H. (1979). Annihilation of dislocations during tensile and cyclic deformation and limits of dislocation densities. Philos. Mag. A.

[33] Brown L.M. (2006). Dislocation bowing and passing in persistent slip bands. Philos. Mag..

